# Literature-Wide Association Studies (LWAS) for a Rare Disease: Drug Repurposing for Inflammatory Breast Cancer

**DOI:** 10.3390/molecules25173933

**Published:** 2020-08-28

**Authors:** Xiaojia Ji, Chunming Jin, Xialan Dong, Maria S. Dixon, Kevin P. Williams, Weifan Zheng

**Affiliations:** BRITE Institute and Department of Pharmaceutical Sciences, North Carolina Central University, Durham, NC 27707, USA; xji@nccu.edu (X.J.); cjin@nccu.edu (C.J.); xdong@nccu.edu (X.D.); maria.m.dixon37@gmail.com (M.S.D.)

**Keywords:** text mining, Word2Vec, rare diseases, IBC, inflammatory breast cancer, drug repurposing

## Abstract

Drug repurposing is an effective means for rapid drug discovery. The aim of this study was to develop and validate a computational methodology based on Literature-Wide Association Studies (LWAS) of PubMed to repurpose existing drugs for a rare inflammatory breast cancer (IBC). We have developed a methodology that conducted LWAS based on the text mining technology Word2Vec. 3.80 million “cancer”-related PubMed abstracts were processed as the corpus for Word2Vec to derive vector representation of biological concepts. These vectors for drugs and diseases served as the foundation for creating similarity maps of drugs and diseases, respectively, which were then employed to find potential therapy for IBC. Three hundred and thirty-six (336) known drugs and three hundred and seventy (370) diseases were expressed as vectors in this study. Nine hundred and seventy (970) previously known drug-disease association pairs among these drugs and diseases were used as the reference set. Based on the hypothesis that similar drugs can be used against similar diseases, we have identified 18 diseases similar to IBC, with 24 corresponding known drugs proposed to be the repurposing therapy for IBC. The literature search confirmed most known drugs tested for IBC, with four of them being novel candidates. We conclude that LWAS based on the Word2Vec technology is a novel approach to drug repurposing especially useful for rare diseases.

## 1. Introduction

There are multiple approaches to identifying a new drug. In a typical drug discovery project, a first-step may be to identify hits via high-throughput screening of compound libraries [1]. In addition, virtual screening methods, including those based on quantitative structure-activity relationship (QSAR) modeling and molecular docking, also play a crucial part in the drug discovery process [2]. These methods require either a training set of a fair number of known compounds and their biological activity against a relevant assay for QSAR modeling or 3D (3-dimensional) structures of drug targets for molecular docking studies, which may not always be feasible in rare disease research. To tackle drug discovery in these difficult situations, a complementary strategy called drug repurposing has been proposed that aims to identify and validate new uses for existing or developmental drugs that are outside the scope of the original medical indication [3,4,5]. In other words, drug repurposing can be defined as finding drugs previously approved by the Food and Drug Administration (FDA) for one disease and using them to treat another. Due to the fast growth of bioinformatics and chemical biology databases, this strategy has become a less risky, more rapid, and lower cost approach compared to traditional drug discovery methods; and it may also reveal new targets and pathways that can be further exploited [4,6]. Just as famously stated by the 1998 Nobel laureate, Sir James Black, that “the most fruitful basis for the discovery of a new drug is to start with an old drug.” [7], drug repurposing strategy has recently been reported to account for 30% of the new drug approvals by the FDA [4].

The main task in drug repurposing is to identify hidden drug-disease relationships. The easy access to various drug and disease databases such as DrugBank [8], ChemBank [9], OMIM [10], KEGG [11], and PubMed [12] has enabled many computational approaches, among which text mining of PubMed is of great potential for rare or understudied diseases [5]. The foundation of text mining for drug repurposing is the recognition that the linguistic context of each biological concept carries critical information and attributes about the underlying biological concept. For example, the context of the word “Tylenol” should reflect the drug’s function as well as related targets, pathways or diseases; thus, “Tylenol” may be associated with latter concepts. A literature-wide association study of such textual data may reveal new knowledge about “Tylenol” and likewise other drugs. Based on these contextual attributes/features, similar drugs related to “Tylenol” could be identified. The same is true regarding different diseases. For example, “pneumonia” may be associated with “influenza” or “cytokine storm”. Thus, drugs used for “cytokine storm” may be useful as a part of the therapeutic approach to certain “pneumonia”. A systematic analysis of the whole biomedical literature (e.g., PubMed) should therefore afford us new information regarding relationships between drugs and diseases. We call this kind of analysis the Literature-Wide Association Study or simply LWAS. Although challenges still remain as to how to most effectively identify novel and meaningful relationships among biological entities via LWAS, progress has been made in recent years as demonstrated by applications from different fields [13,14,15,16], among which Word2Vec has turned out to be a powerful technology [17].

Word2Vec was first developed by Mikolov et al. [17]. It creates models based on a two-layer neural network that is trained to reconstruct linguistic contexts of words in a textual corpus. Briefly, a large textual corpus (e.g., PubMed abstracts) is provided to the program; it then produces word vectors of several hundred dimensions for all detected words in the corpus. The words are positioned in the vector space such that those with common linguistic context are found in close proximity. It has been employed for drug repurposing in a couple of cases [18,19].

We are interested in studying inflammatory breast cancer (IBC), which is a highly aggressive and lethal form of breast cancer [20]. Although it only represents about one to five percent of breast cancers [21], it is estimated to account for ten percent of breast cancer deaths in the USA [22]. Diagnosis of IBC is difficult as it currently relies on clinicopathologic features with IBC tumors not easily detected by mammogram [20]. Outcomes for IBC patients are worse in comparison to non-IBC breast cancer patients [23]. Standard treatment for IBC includes neoadjuvant chemotherapy, followed by mastectomy and then radiation therapy and is summarized in [24]. Depending on subtype, treatment may include HER2 targeted therapy or hormone therapy. Treatment options for triple negative (TN) IBC are limited [25]. It has been challenging to identify molecular targets unique to IBC and no therapeutics have been developed that target IBC specifically [26,27].

The aim of this study was to develop and validate an in silico methodology based on LWAS of PubMed to propose existing drugs for IBC. Over 3.80 million “cancer”-related PubMed abstracts were processed and used as the textual corpus for developing the Word2Vec embedding models. The resulting embedding vectors for drugs and diseases established quantitative representation of the drugs and diseases. The contextual similarity among these drugs and diseases can be quantified based on the semantic similarity among the word vectors. In this study, 336 known drugs and 370 diseases were represented by Word2Vec-derived embedding vectors. Information on 970 known drug-disease pairs among these drugs and diseases collected from DrugBank [8] and KEGG [11] was used as the basis set for establishing new relationships among drugs and diseases. The analysis has resulted in 18 seemingly unrelated diseases that were similar to IBC, with 24 corresponding FDA-approved drugs. Interestingly, IBC did not cluster with non-IBC breast cancer. These 24 drugs were proposed as potential candidates for treating IBC, which was further supported by a comprehensive literature review that showed plausible relationships between the proposed drugs and IBC.

## 2. Results

### 2.1. PubMed Abstracts on “Cancer” Collection

A search of the PubMed database with “cancer” as the search term identified 3,799,365 items at that time point. The search traced back to the first record in 1787. A total of 28,461 items were published in the first 64 years from 1787 to 1950. Between 1981–1990, the number of publications reached 427,584 in ten years, averaging 42,758 per year. Starting 1991, the average publications per year was about 50,000. In 2005 alone, however, about 100,000 publications were found to include the key word “cancer”. Since 2015, more than 250,000 items per year have been found. The hidden knowledge could be huge since no one is equipped to read such an enormous amount of publications and make relevant connections without formal analysis with the aid of computational algorithms. All abstracts were downloaded and processed using the R package, easyPubMed [28], to extract the “abstract” content.

### 2.2. Corpus Preparation and Word Embedding

After text corpus preparation according to the protocol (cf. Methods), the above body of abstracts produced 3,466,932 sentences. These sentences were used as the input to the word embedding program Word2Vec, which produced word vectors for all the terms (i.e., the vocabulary) in the corpus. The Gensim implementation of Word2Vec [29] was used in this study, with the following parameters: sentences = 1, vector size = 150, window size = 5, minimum count of words to be embedded = 1 (i.e., all words), workers = 1, skip-gram= 0.

As a result, 791,486 words in the previous 3,466,932 sentences were embedded into vectors, each corresponding to a unique term. For example, the disease “IBC” was represented by a 150-dimensional vector; so was the meningioma drug “imatinib”.

We identified 1,121 FDA-approved drug-disease pairs from the combined sources of DrugBank [8] and KEGG [11], which included 346 unique drugs and 434 unique diseases (Supplementary Materials Appendix 1–3). Out of the 346 unique drugs, 336 were in the above embedding model, producing 336 150-dimensional drug vectors (Supplementary Materials Appendixes 4 and 5). Out of the 434 unique diseases, 370 were found in the above word embedding model, producing 370 150-dimensional disease vectors (Supplementary Materials Appendixes 6 and 7). As a result, there were 970 drug-disease pairs with corresponding embedding vectors (Supplementary Materials Appendix 8).

### 2.3. Prediction of Drugs for IBC Based on Drug Similarity and Disease Similarity Analyses

**Figure 1:**
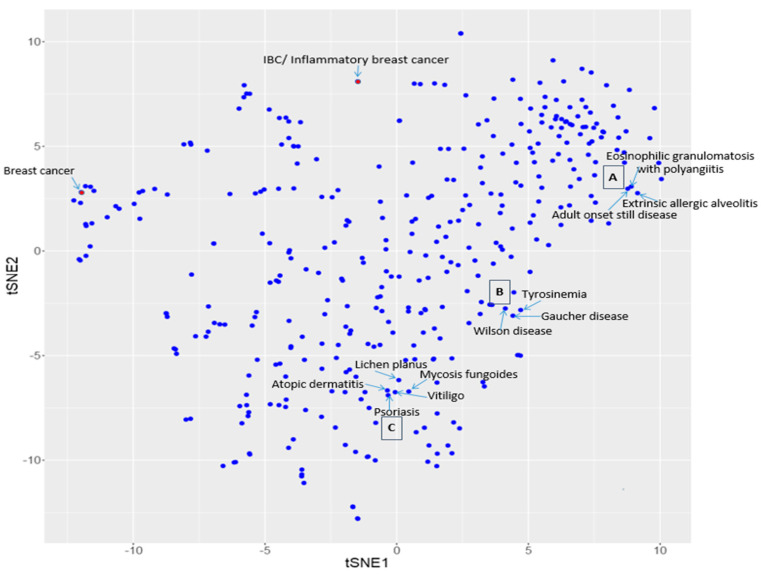
Breast cancer, inflammatory breast cancer (IBC), and three annotated disease clusters on the disease similarity map.

**Figure 2:**
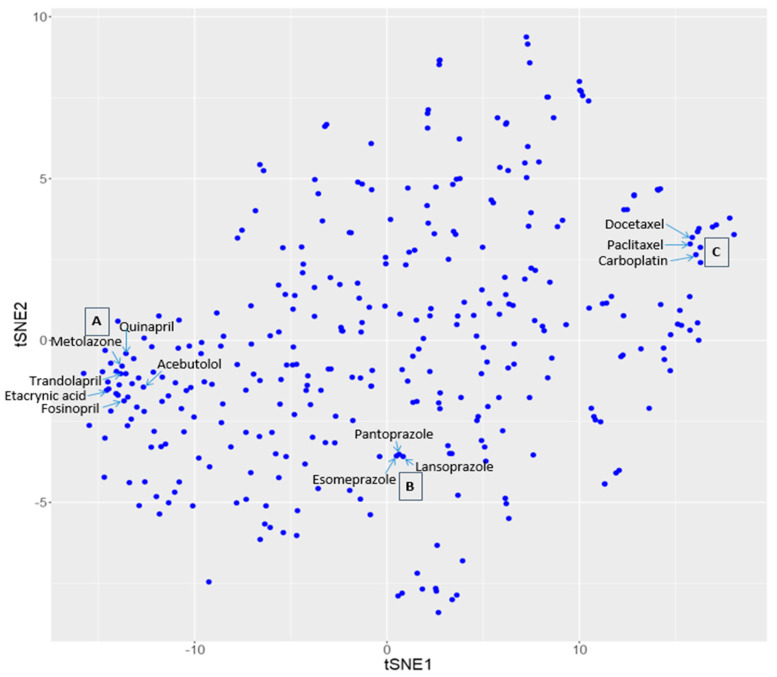
Three clusters of drugs on the drug similarity map.

The t-SNE technology was employed to map drugs and diseases on two separate 2-dimensional (2D) scatter plots. Figure 1 is the disease-disease similarity map by projecting the 150-dimensional vectors onto a 2D scatter plot, each point corresponding to one of the 370 diseases. Similarly, Figure 2 is the drug-drug similarity map by projecting the 336 drug vectors onto a 2D scatter plot.

Based on the similarity principle, similar diseases and similar drugs should be clustered in proximity on their respective similarity map. Figure 1 and Figure 2 showed the examples of clustered diseases and clustered drugs, respectively. In Figure 1, it can be seen that cluster A contains three immune system/inflammatory diseases. They are adult onset still disease (H01516), eosinophilic granulomatosis with polyangiitis (H01468), and extrinsic allergic alveolitis (H00346). Cluster B contains three inherited metabolic and nervous system diseases. They are Gaucher disease (H00126), tyrosinemia (H00165), and Wilson disease (H00210). Cluster C contains five skin and connective tissue disease and immune system diseases: atopic dermatitis (H01350), mycosis fungoides (H01463), psoriasis (H01656), and vitiligo (H01372). In Figure 2, it can be seen that cluster A contains six high blood pressure (H01633) drugs: Quinapril (DB00881), Metolazone (DB00524), Trandolapril (DB00519), Ethacrynic acid (DB00903), Fosinopril (DB00492), and Acebutolol (DB01193). Cluster B contains three eosinophilic esophagitis (H01361) drugs: Esomeprazole (DB00736), Lansoprazole (DB00448), and Pantoprazole (DB00213). Cluster C contains three breast cancer (H00031) drugs: Docetaxel (DB01248), Paclitaxel (DB01229), and Carboplatin (DB00958). Note that the ID numbers following the disease names are KEGG ID’s for diseases; and the ID numbers after drug names are the drug ID’s in the Drug Bank. These examples demonstrate that similar diseases and similar drugs are indeed clustered in proximity on the respective t-SNE-derived scatter plots.

**Figure 3:**
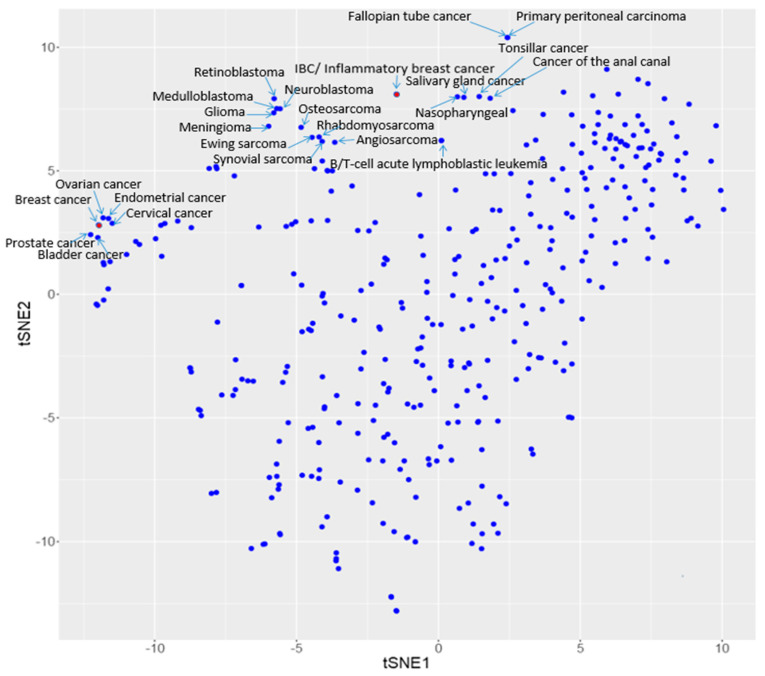
Diseases clustered with IBC and breast cancer, respectively, on the disease similarity map.

Based on the above observation, we hypothesized that diseases similar to IBC should be clustered in proximity on the disease similarity map, and the corresponding similar drugs should be clustered in proximity on the drug similarity map. Diseases similar to IBC (our target disease) are shown in Figure 3 and Table 1. Not surprisingly, IBC and inflammatory breast cancer overlap on Figure 3. Eighteen (18) diseases were found in the vicinity of IBC on this disease similarity map. They belong to several different categories: (1) cancers of the eye, brain, and central nervous system include retinoblastoma (H01513), neuroblastoma (H00043), medulloblastoma (H01667), glioma (H00042), and meningioma (H01556); (2) cancers of soft tissues and bone include osteosarcoma (H00036), Ewing sarcoma (H00035), rhabdomyosarcoma (H00037), synovial sarcoma (H00050), and angiosarcoma (H01666); (3) cancers of haematopoietic and lymphoid tissues include B-cell acute lymphocytic leukemia (H00001) and T-cell acute lymphocytic leukemia (H00002); (4) head and neck cancers include nasopharyngeal cancer (H00054), salivary gland cancer (H01508), and tonsillar cancer (H01509); (5) cancer of the digestive system include the cancer of the anal canal (H00044); (6) cancers of the breast and female genital organs include fallopian tube cancer (H01554) and primary peritoneal carcinoma (H01665).

**Figure 4:**
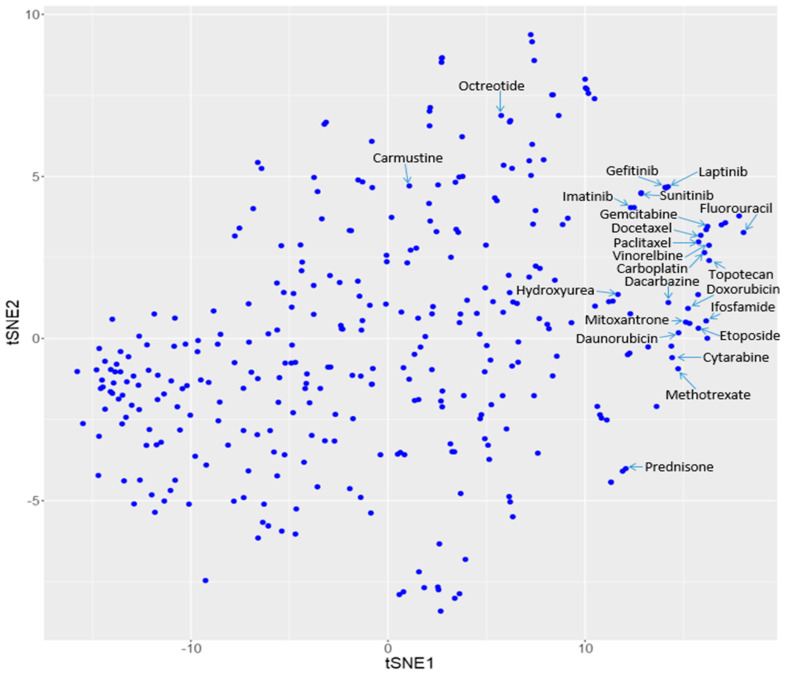
Text mining predicted drugs for IBC on the drug similarity map.

We searched the reference dataset of 970 known disease-drug pairs for drugs corresponding to the aforementioned diseases similar to IBC. Twenty four (24) unique drugs were found for the above 18 diseases: Carboplatin (DB00958), Carmustine (DB00262), Cytarabine (DB00987), Dacarbazine (DB00851), Daunorubicin (DB00694), Docetaxel (DB01248), Doxorubicin (DB00997), Etoposide (DB00773), Fluorouracil (DB00544), Gefitinib (DB00317), Gemcitabine (DB00441), Hydroxyurea (DB01005), Ifosfamide (DB01181), Imatinib (DB00619), Lapatinib (DB01259), Methotrexate (DB00563), Mitoxantrone (DB01204), Octreotide (DB00104), Paclitaxel (DB01229), Prednisone (DB00635), Sunitinib (DB01268), Topotecan (DB01030), Vincristine (DB00541), Vinorelbine (DB00361). It can be seen that these drugs were clustered in proximity on the drug similarity map (Figure 4 and Table 2).

### 2.4. Literature Validation

We have searched the biomedical literature for relevant information in order to evaluate whether the aforementioned predicted drugs could indeed have potential against IBC. Listed in Table 3 are the 24 predicted drugs, with their names and whether or not they had been reported in PubMed, ClinicalTrials.gov, or both. Nineteen (19) of them have PubMed support, meaning they were studied in various IBC models according to PubMed publications. Eleven (11) of the 24 drugs with different mechanisms of action had been tested in clinical trials with varying degrees of clinical benefits for IBC [30,31,32,33,34,35,36,37]. This indicates that our prediction has literature support. A number of studies have been performed to identify the best agents to use for neoadjuvant chemotherapy in IBC and the findings as of 2015 were summarized in [38]. Eleven drugs had been tested in clinical trials for IBC and were reported in ClinicalTrials.gov [39], among which four are FDA-approved drugs for breast cancer treatment. Among those in the ClinicalTrials.gov database, Sunitinib was not reported in the PubMed literature, and it is a prediction from our LWAS method. Four additional drugs were not found in PubMed; neither had they been tested in clinical trials for IBC in ClinicalTrials.gov. These four drugs are considered to be our prediction of novel candidates for IBC.

## 3. Discussion

### 3.1. Disease-Disease Similarity Analysis

It is interesting that, on Figure 3, IBC is closer to the aforementioned 18 diseases rather than breast cancer (H00031), which was clustered with ovarian cancer (H00027), endometrial cancer (H00026), cervical cancer (H00030), prostate cancer (H00024), and bladder cancer (H00022). The clustering of “breast cancer” with other female gynecological cancers on the disease similarity map including ovarian, endometrial, bladder, and cervical cancers could be expected—previous studies suggested that women with breast cancer had higher incidence of developing these secondary gynecological malignancies [40]. Without human intervention, our LWAS method has successfully grouped these diseases together. Interestingly, the LWAS analysis also revealed the clustering between breast cancer and prostate cancer, which may be due to a strong family history or carriers of genetic mutations found in patients of both cancers [41]. Other factors such as hormone-status, environment, and lifestyle are common risk factors of these reproductive cancers in men and women [42,43]. It was interesting that IBC did not cluster with breast cancer on the disease similarity map. This finding is consistent with the known distinctiveness of this form of breast cancer. It is known that patients with IBC have unique clinical features including tumor emboli in the dermal lymphatics of the breast instead of solid tumors [44,45]. This finding based on LWAS that IBC and breast cancer cluster differently was also consistent with a recent review that provided an overview of the unique clinical and molecular characteristics of IBC, and that IBC should be considered as a separate entity from non-IBC breast cancer [26].

### 3.2. Drug Candidates for Repurposing

The combined similarity analyses and reference dataset comparison afforded interesting repurposing candidates for validation and follow-up experiments. Current IBC treatment includes the use of anthracycline-based chemotherapy and a taxane-based chemotherapy. As positive controls we would expect to identify these types of drugs in our analysis; indeed, we have found such examples. As IBC was clustered close to primary peritoneal carcinoma (H01665), drugs used to treat “primary peritoneal carcinoma” should potentially work for IBC. It is known that Carboplatin and Paclitaxel are the chemotherapies most often used for primary peritoneal carcinoma [46]. Indeed, Paclitaxel (Taxol), which is a taxane, is part of the standard neoadjuvant chemotherapy regime for IBC, although the use of Carboplatin as a standard chemotherapy for IBC still lacks consensus [24]. Furthermore, Doxorubicin, another one of the 24 drugs identified, is an anthracycline and was also endorsed as a standard treatment for IBC [24]. These facts appeared to support the discovery from our study.

### 3.3. Literature Validation

We have searched the biomedical literature for relevant information in order to further evaluate whether the predicted drugs could indeed have potential against IBC. Among the 24 predicted drugs, 19 of them (Table 3) have PubMed support, i.e., they were studied in various IBC models. Eleven of the 24 drugs with different mechanisms of action (DNA damage, neuronal signaling, protein tyrosine kinase, and cytoskeletal signaling) have been tested in clinical trials with varying degrees of clinical benefits for IBC [30,31,32,33,34,35,36,37]. A number of studies have been conducted to identify the best agents to use for neoadjuvant chemotherapy in IBC and the findings as of 2015 are summarized in [38]. Sunitinib, Lapitib, kinase inhibitors, and Vinorelbine, Fluorouracil and Methotrexate have been tested in trials including IBC patients. These results appear to suggest that our methodology was effective in finding drugs that have potential against IBC. However, these papers had been published before 2019 (i.e., covered in the corpus used for Word2Vec analysis). Therefore, this could be considered as a self-validation, indicating that LWAS based on Word2Vec had indeed captured historic knowledge. Eleven drugs (Carboplatin, Docetaxel, Doxorubicin, Etoposide, Fluorouracil, Gemcitabine, Lapatinib, Methotrexate, Paclitaxel, Sunitinib, and Vinorelbine) were tested in clinical trials for IBC and were reported in ClinicalTrials.gov [39], among which four drugs (Carboplatin, Docetaxel, Doxorubicin, and Paclitaxel) have been FDA-approved for breast cancer treatment. Among those in the ClinicalTrials.gov database, Sunitinib was not reported in the PubMed literature, and thus not included in our training corpus (Table 3). This example showed that the LWAS methodology did correctly predict this drug to be useful for IBC, even though PubMed had not reported it. This is a great example of the potential predictiveness of the LWAS method.

Four additional drugs (Cytarabine, Dacarbazine, Hydroxyurea, and Topotecan) were not found in the PubMed literature; neither had they been tested in clinical trials for IBC in ClinicalTrials.gov. As a logical step, one may use molecular modeling tools, such as docking or pharmacophore matching, to compare the proposed drug candidates against the molecular model (X-ray or homology model) of a specific target for IBC. However, in the case of IBC (a rare disease), the situation precludes such an approach. Some biomarkers are implicated in IBC, but no single molecular feature or genetic alteration is sufficient to identify IBC as a distinct type of breast cancer [26]. Some biomarkers have been identified as strongly associated with IBC, in particular, elevated expression of the adhesion protein E-cadherin is a hallmark of IBC [47]. Other IBC-linked biomarkers include overexpression of the translation factor eIF4G [48] and elevated expression of RhoC GTPase [49]. Elevated JAK/STAT pathway activity [50], in particular increased phospho-STAT3 and JAK2, is seen in IBC compared to non-IBC. But no single protein has been validated as the disease target for IBC.

We have examined the mechanisms and disease indications of the four proposed drug candidates (Cytarabine, Dacarbazine, Hydroxyurea, and Topotecan) in DrugBank [8]. Cytarabine is a pyrimidine nucleoside analog, an antimetabolite anticancer agent that inhibits the synthesis of DNA. It is used mainly in the treatment of leukemia. Hydroxyurea also inhibits DNA synthesis through the inhibition of ribonucleoside diphosphate reductase. It is used for melanoma, resistant chronic myelocytic leukemia, and recurrent, metastatic, or inoperable carcinoma of the ovary. Dacarbazine has significant activity against melanomas; and the mechanism of action is not yet clear, but appears to exert cytotoxic effects via its action as an alkylating agent. Another hypothesis is that it may inhibit DNA synthesis by its action as a purine analog. Finally, Topotecan is used to treat ovarian cancer. It works by inhibiting DNA topoisomerases I.

Even though the above four drugs have not yet been shown to affect the specific biomarkers implicated in IBC, they are involved in several common mechanisms of anticancer agents, i.e., inhibition of DNA synthesis, alkylation, or topoisomerase inhibition. Thus, these four drugs may have applications for a variety of cancers including IBC. In the future, we plan to test all 24 predicted drugs in IBC tumor spheroid models and a subset of them will be tested in vivo models [51,52].

## 4. Methods

### 4.1. The Overall Design

**Figure 5:**
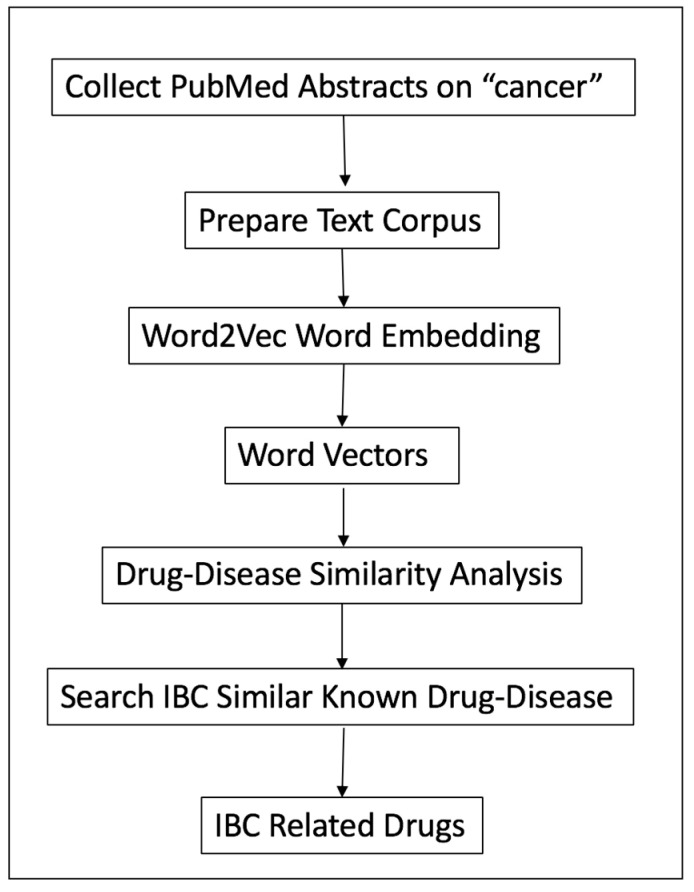
The Literature-Wide Association Studies (LWAS)-IBC drug repurposing workflow.

The overall design of the LWAS procedure for drug repurposing is shown in Figure 5. The 3.8 million textual abstracts from PubMed were downloaded and pre-processed for the Word2Vec algorithm to build embedding models. Once an embedding model was built, using specific parameters, all the vectors representing respective biological terms, namely drug names and disease names, were obtained. The similarity among drugs and diseases was analyzed using the t-Distributed Stochastic Neighbor Embedding (t-SNE) mapping technology [53], from which similar diseases to IBC were identified as well as their corresponding drugs based on a list of known drug-disease pairs collected in this study as the reference set. The individual components are now detailed as follows.

### 4.2. Collecting and Preprocessing PubMed Abstracts

A raw corpus of 3.8 million PubMed abstracts, covering the years from 1787 to 2019, was downloaded in XML format with the keyword “cancer” as the filter. An R program was used to extract the “abstract” text for further processing below.

### 4.3. Preparing the Text Corpus

Abstracts were cleaned by removing all punctuations and converting all letters to uppercase for consistency. Some drug and most disease names consist of two or more words. These multiword terms were converted into single words—the spaces between all words of the multiword drug names and disease names were replaced with a hyphen “-”. As a result, these names were treated as hyphenated single words in the vocabulary of the Word2Vec model.

### 4.4. Performing Word Embedding

Word2Vec processes a text corpus and creates numerical representation of each word in the corpus. In other words, its input is a text corpus (i.e., PubMed abstracts in this study) and its output is a set of vectors that represent all the words (i.e., vocabulary) occurring in that corpus. The objective of the Word2Vec algorithm is to generate these vectors so that similar words occur in proximity in a high dimensional space. Similarity of words is based on the context of individual words—words with similar textual context will be given similar vectors. Given enough data it can make highly accurate predictions about a word’s meaning based on its past appearances in the corpus. It can establish a word’s association with other words. Compared to cheminformatics, a word vector created by Word2Vec is like a vector formed by molecular descriptors for a given molecule based on its molecular features. Just like two molecules with similar features will have similar vectors of molecular descriptors, two words with similar linguistic context (i.e., word features) will be given similar vectors by Word2Vec model. As in cheminformatics, clustering analysis or multidimensional scaling can then be used to group or visualize molecules to detect similarity patterns, the same tools can be used to analyze Word2Vec generated vectors to discover similarity among words (e.g., drug names and disease names).

Technically speaking, let *Cw* represent the textual context of a given word *w*, which is the set of words surrounding the word *w* within a textual window (e.g., +/−5 or +/−10 words). The algorithm is to find a function or a model *f* so that it can accurately predict *Cw* from *w*: *f*(*w*) → *Cw* for all the words in the vocabulary found in the corpus. For a large text corpus, the function can be learned or approximated via a two-layer neural net as developed by Mikolov [17]. The Gensim implementation [29] of the Word2Vec algorithm was used in this analysis. In this study, 150-dimensional vectors were generated for the biological terms (drugs and diseases) in the vocabulary of the PubMed corpus. These word vectors were analyzed to generate drug similarity and disease similarity maps as follows.

### 4.5. Generating Drug Similarity and Disease Similarity Maps

To visualize the similarity relationships among drugs and diseases, t-SNE [53] was employed to map the word vectors from high dimensional space onto a 2-dimensional (2D) space, creating 2D scatter plots. In this study, drug vectors and disease vectors were mapped onto a drug similarity map and a disease similarity map, respectively. The disease similarity map was used to discover similar diseases to IBC, and the associated similar drugs were discovered on the drug similarity map based on a reference set of known drug-disease pairs detailed below.

### 4.6. Preparing a Reference Set of Known Drug-Disease Pairs

DrugBank [8] provides critical information of FDA-approved drugs and their associated diseases. By combining this information and that identified from the KEGG database [11], we identified over 1000 pairs of drugs and diseases. This set of drug-disease pairs connects the drug space and the disease space, and established relationships among certain drugs and diseases. This information will serve as the basis for identifying drugs associated with diseases that are similar to IBC. These identified drugs were hypothesized to be the repurposing candidates for IBC.

## 5. Conclusions

The Literature-Wide Association Study (LWAS) based on Word2Vec technology is a plausible approach to drug repurposing for rare or understudied diseases. This study, to the best of our knowledge, is the first to employ this method to mine the PubMed literature for IBC drugs. In a retrospective literature analysis, we have demonstrated that this method could find relevant drugs, not only those that had been tested in the past as internal validation of the approach, but also drugs that were not yet tested but mechanistically reasonable for prospective testing in future experimental studies. Of note is that this text mining approach can be employed to discover potential drug repurposing candidates even if the target of the disease is not known, which is of special value for rare disease drug repurposing.

With new experimental data that will be collected in the course of future studies, we will validate and refine the word embedding models. In addition, novel machine learning techniques such as those implemented in the Scikit-learn package [54] will be employed to analyze the new data. We will also perform drug similarity analysis based on chemical descriptors (rather than Word2Vec embeddings alone) of the drugs, which can further expand the approach to enable the discovery of new chemical entities (NCE) beyond drug repurposing for IBC. This combined text mining and cheminformatics approach should afford a great opportunity for uncommon diseases such as IBC and other rare diseases where new therapeutic discoveries are greatly needed.

On a technical level, we have currently collected 1121 drug-disease pairs involving FDA-approved drugs from the DrugBank and KEGG databases. The continuing updates and expansion of these two databases provide an opportunity for further expanding the reference set of drug-disease pairs. This will be implemented in the future version of LWAS and should further enhance this methodology. We also note that the software used herein has been previously published, verified, and is open-source; thus, this study provides a general-purpose protocol that can be applied to drug repurposing for other cancers, and indeed for other diseases as well.

## Figures and Tables

**Table 1:** Diseases clustered close to IBC and their corresponding drugs.

Disease ID (KEGG)	Disease Name	Drugs
H01513	Retinoblastoma	carboplatin, etoposide, vincristine
H00043	Neuroblastoma	doxorubicin, etoposide
H01667	Medulloblastoma	carboplatin, topotecan, vincristine
H00042	Glioma	carmustine, vincristine
H01556	Meningioma	hydroxyurea, imatinib, octreotide
H00036	Osteosarcoma	doxorubicin, ifosfamide, methotrexate
H00035	Ewing sarcoma	dacarbazine, doxorubicin, etoposide, ifosfamide, vincristine
H00037	Rhabdomyosarcoma	dacarbazine, doxorubicin, ifosfamide, imatinib, sunitinib
H00050	Synovial sarcoma	dacarbazine, doxorubicin, ifosfamide, imatinib, sunitinib
H01666	Angiosarcoma	doxorubicin, ifosfamide, paclitaxel
H00001	B-cell acute lymphocytic leukemia	cytarabine, daunorubicin, doxorubicin, methotrexate, prednisone, vincristine
H00002	T-cell acute lymphocytic leukemia	cytarabine, daunorubicin, doxorubicin, methotrexate, prednisone, vincristine
H00054	Nasopharyngeal cancer	carboplatin, docetaxel, fluorouracil, paclitaxel
H01508	Salivary gland cancer	gefitinib, gemcitabine, imatinib, lapatinib, mitoxantrone, paclitaxel, vinorelbine
H01509	Tonsillar cancer	carboplatin, docetaxel, fluorouracil, paclitaxel
H00044	Cancer of the anal canal	doxorubicin, fluorouracil
H01554	Fallopian tube cancer	carboplatin, paclitaxel
H01665	Primary peritoneal carcinoma	carboplatin, paclitaxel

**Table 2:** Predicted drugs for IBC.

Drug ID (Drug Bank)	Drug Name
DB00958	Carboplatin
DB00262	Carmustine
DB00987	Cytarabine
DB00851	Dacarbazine
DB00694	Daunorubicin
DB01248	Docetaxel
DB00997	Doxorubicin
DB00773	Etoposide
DB00544	Fluorouracil
DB00317	Gefitinib
DB00441	Gemcitabine
DB01005	Hydroxyurea
DB01181	Ifosfamide
DB00619	Imatinib
DB01259	Lapatinib
DB00563	Methotrexate
DB01204	Mitoxantrone
DB00104	Octreotide
DB01229	Paclitaxel
DB00635	Prednisone
DB01268	Sunitinib
DB01030	Topotecan
DB00541	Vincristine
DB00361	Vinorelbine

**Table 3:** Evaluation of predicted IBC drugs in PubMed or ClinicalTrials.gov.

Predicted Drugs for IBC	PubMed	ClinicalTrials.gov
Carboplatin	Yes	Yes
Carmustine	Yes	No
Cytarabine	No	No
Dacarbazine	No	No
Daunorubicin	Yes	No
Docetaxel	Yes	Yes
Doxorubicin	Yes	Yes
Etoposide	Yes	Yes
Fluorouracil	Yes	Yes
Gefitinib	Yes	No
Gemcitabine	Yes	Yes
Hydroxyurea	No	No
Ifosfamide	Yes	No
Imatinib	Yes	No
Lapatinib	Yes	Yes
Methotrexate	Yes	Yes
Mitoxantrone	Yes	No
Octreotide	Yes	No
Paclitaxel	Yes	Yes
Prednisone	Yes	No
Sunitinib	No	Yes
Topotecan	No	No
Vincristine	Yes	No
Vinorelbine	Yes	Yes

## Supplementary Materials

The following are available online. Appendix Files: Appendix 1_1121_Drug-Disease _Pairs. Appendix 2_346_Unique_Drugs. Appendix 3_ 434_Unique_Diseases. Appendix 4_Word2Vec_Trained_336_Drugs. Appendix 5_Word2Vec_336_Drug_Vectors. Appendix 6_Word2Vec Trained_370 Diseases. Appendix 7_Word2Vec_370_Disease_Vectors. Appendix 8_970_Trained_Drug-Disease_Pairs.

## Abbreviations

FDA	Food and Drug Administration
IBC	Inflammatory Breast Cancer
LWAS	Literature-Wide Association Studies
NCE	new chemical entities
QSAR	quantitative structure-activity relationship
TN	triple negative
t-SNE	t-Distributed Stochastic Neighbor Embedding
